# Dietary habits, food taboos, and perceptions towards weight gain during pregnancy in Arsi, rural central Ethiopia: a qualitative cross-sectional study

**DOI:** 10.1186/s41043-016-0059-8

**Published:** 2016-07-25

**Authors:** Taddese Alemu Zerfu, Melaku Umeta, Kaleab Baye

**Affiliations:** 1 Center for Food Science and Nutrition, Addis Ababa University, College of Natural Sciences, Addis Ababa, Ethiopia; 2 Department of Medical Biochemistry, Addis Ababa University, College of Health Sciences, School of Medicine, Addis Ababa, Ethiopia

**Keywords:** Food taboo, Maternal nutrition, Dietary diversity, Pregnancy, Weight gain

## Abstract

**Background:**

The nutritional status of women before and during pregnancy can be determined by maternal knowledge, attitudes, and perceptions towards certain foods. The present study aimed to explore maternal dietary habits, food taboos, and cultural beliefs that can affect nutrition during pregnancy in rural Arsi, central Ethiopia.

**Methods:**

A qualitative, cross-sectional study, involving 38 key informant in-depth interviews and eight focus group discussions, was conducted among purposefully selected pregnant women and their husbands, elderly people, community leaders, health workers, and agriculture office experts. Participants were selected purposefully from all the major agro-ecologic areas of the study site. Data was analyzed manually using the thematic framework analyses method.

**Results:**

The pregnant women reported that they did not change the amount and type of foods consumed to take into account their increased nutritional need during pregnancy. The consumption of meat, fish, fruits, and some vegetables during pregnancy remained as low as the pre-pregnancy state, irrespective of the women’s income and educational status. Although not practiced by all, a number of taboos related to the intake of certain food items and misconceptions that can adversely affect nutritional status during pregnancy were identified. The most common taboos were related to the consumption of green leafy vegetables, yogurt, cheese, sugar cane, and green pepper. However, the frequency and extent of the practice varied by maternal age, family composition, and literacy level. Older mothers, from rural villages, and those with no formal education were more likely to practice the taboos than younger and educated ones. Almost all of the participants disfavored weight gain during pregnancy in fear of obstetric complications associated with the delivery of a bigger infant.

**Conclusions:**

Misconceptions about weight gain during pregnancy and food taboos were widespread, particularly among older and illiterate rural communities. Thus, future nutrition programs should promote diversification of both the agricultural production and consumption.

## Background

Access to safe, adequate, and nutritious food is a basic human right that is essential for good health [1, 2]. Unfortunately, this basic right is denied in many low- and middle-income countries (LMIC) partly due to food insecurity, poverty, and inappropriate food distribution [3]. Consequently, children, women of reproductive age, pregnant, and lactating women are disproportionately affected by under nutrition and its associated adverse effects [4]. Low dietary intakes, inequitable intra-household food distribution, recurrent infections, and poor care in general are among the leading causes of under nutrition, but food taboos and misconceptions can also contribute significantly to the high levels of under nutrition [5, 6].

Food taboos refer to the restriction of specific foods as a result of social or religious customs. In many traditional societies, cultural norms and customs govern behaviors including during critical life stages like pregnancy [7]. Pregnancy is a particular period when physiological nutrient demands are substantially increased. To meet this increased nutrient requirement for both the woman and the fetus, a pregnant woman is supposed to increase the amount and quality of foods she consumes [8, 9]. Nevertheless, when misconceptions or food taboos exist, the pregnant woman’s ability to meet such increased demands can even be more compromised, hence putting the woman at a greater risk of adverse pregnancy outcomes [10].

Various forms of taboos, misconceptions, and cultural beliefs towards certain foods exist in various countries [11]. For example, foods consumed cold like fruits and vegetables were reported to be taboo among nursing mothers in Mexico [12]. Similarly, snails and grass-cutter meat are taboo among pregnant women and eggs among children in South Eastern Nigeria [13]. To what extent such food taboos and misconceptions exist and how they affect pregnancy outcomes in Ethiopia remain largely unknown.

This is unfortunate, since such information could be considered in the design of nutrition interventions targeting pregnant women. Besides, studies that involve a number of actors that can influence food consumption like health workers, the elderly, and agricultural extension workers, as well as husbands of pregnant women are rare, if not non-existent. Therefore, the present study used focus group discussions (FGDs) with pregnant women and their husbands, as well as key informant interviews (KIIs) with various health, agriculture, and community leaders to explore maternal dietary habits, food taboos, and misconceptions that can affect nutrition during pregnancy in rural Ethiopia.

## Methods

### Study site

The study was conducted in 12 rural villages selected from four districts of Arsi Zone, central part of the Oromia Regional State, Ethiopia. The Zonal capital, Assela, is located at 175 km from Addis Ababa and has a total area of 210,082 km^2^ [14]. The study sites (12 villages) were randomly selected from four rural districts that were selected purposefully.

### Study participants and design

The study employed a cross-sectional qualitative study design, mainly KIIs and FGDs. Since food taboos and dietary habits are sensitive issues, they are best explored through qualitative methods like FGDs and KIIs rather than quantitative methods. Both FGDs and KIIs were used to triangulate individual and group-level opinions. Data were collected between October, 2014 and May, 2015.

We have conducted a total of 38 KIIs and eight FGDs (*n* = 6–10) with subjects selected purposively from rural villages. The FGDs were held with pregnant women and their husbands, separately. The reason for conducting the FGDs with male and female separately was to facilitate the expression of opinions without fear of being judged by their respective partners. Individual KIIs were conducted with health workers, agricultural experts, and the elderly (grandmothers and fathers). The KIIs were held with health workers because they are mainly responsible for the delivery of nutrition education messages to the community; agricultural experts because they can provide information related to the production and availability of certain foods; and the elderly to investigate whether or not a shift away from food-taboo-related practices is present among younger pregnant women. The KIIs were conducted in an interactive manner, whereby the study participants were encouraged to take an active role in establishing the flow of the interview. FGDs and KIIs were held at the nearby health posts or health centers.

Open-ended questions were used to collect relevant information. Questions with redundant responses were considered to be saturated and were removed every evening after transcribing the day’s work and preliminary analysis. New questions were added whenever an information gap was identified. These included questions about knowledge, experience, and opinion about food consumption during pregnancy. Data were collected by two of the research team members and interviews were tape-recorded.

### Data analyses

Data were analyzed manually by applying the thematic framework analysis method [15]. Preliminary manual analysis was an inherent part of the data collection. As more data emerged from everyday encounters, the meaning of certain ideas and concepts were grouped into previously identified categories.

The lead author coded all the transcripts, and all the authors independently read the material and contributed by negotiating the final categories and their contents. Each audiotape interview was professionally transcribed word by word in *Afan Oromo* (local language) and then translated to English.

### Ethics

Ethical clearance was obtained from the Ethics committees of the Addis Ababa University, College of Natural Sciences and the Oromia regional health bureau. Written consent was obtained from each respondent prior to data collection. Privacy and confidentiality were maintained throughout the study.

## Results

We have conducted a total of 38 KIIs and eight FGDs in four districts of Arsi Zone, Oromia region, Ethiopia. Twenty three of the key informants were elderly (grandparents) and six were community leaders, while the remaining nine were health professionals. The participants represented a wide age range (23–92 years). The individual KIIs took 30 min on average, whereas the FGDs with the pregnant women and the husband, each took about an hour and half (Table 1). The findings of the FGDs are summarized as follows.

**Table 1:** Socio-demographic and basic data of qualitative study participants, rural Arsi Ethiopia, 2015

Socio-demographic characteristic	KII (*n* = 38)	FGD (*n* = 59)
	*N* (%)	*N* (%)
Age		
• 23–30	6 (15.8)	*18 (30.5)*
• 31–40	2 (5.3)	17 (28.8)
• 41–50	2 (5.3)	15 (25.4)
• 51–60	1 (2.6)	7 (11.9)
• 60+	*27 (71)*	2 (3.4)
Occupation/role in the community		
• Health worker	9 (23.7)	
• Grand parent	*23 (60.5)*	
• Community leader	6 (15.8)	
Role in the family		
• Wife		*33 (55.9)*
• Husband		26 (44.1)
Sex		
• Male	*21 (55.3)*	*33 (55.9)*
• Female	17 (44.7)	26 (44.1)
Total	38 (100)	59 (100)

Footnote: The maximum frequency (%) under a given category of socio-demographic characteristic is italised for emphasis

### Dietary habits

#### Food types consumed

The diet of the pregnant women was predominantly composed of cereals and legumes that were consumed in the form of *injera* (fermented pancake-like flat bread) and legume-based stews like *shiro*. The food consumption of the pregnant women, like that of the rest of the family, was mainly determined by seasonal variations and the households’ agricultural production.

…we (pregnant women) eat what we get at home,… we mainly produce maize, barley and beans. We eat injera and shiro (local sauce prepared from beans/peas)….., but during ‘belg’(short-rainy season) and ‘kiremt’ (main rainy season), we also harvest and consume potato, cabbage and other vegetables…

Pregnant women, 35 years, FGD participant.

The consumption of fruits and vegetables was reported to be rare. The participants reported that they produce little fruits and vegetables. This in turn limited the availability of fruits and vegetables in the market, further restraining the diets’ diversity.

… my wife is pregnant and I know that she needs to eat a variety of foods including fruits and vegetables … but in our village, we do not produce fruits and thus do not consume them…;if we want to consume fruits, we need to buy them from the district town or the zonal capital, Assela…

Husband of a pregnant woman, 42 years, FGD participant from the highlands.

Nevertheless, some pregnant mothers from the lowland and warm agro-climatic areas reported to have little, and mostly seasonal productions of fruits like papaya, banana, and mangoes. However, these are often destined for the market and not for household consumption.

Agricultural production seems to be a key determinant of consumption and is dependent on seasonal variations and agro-ecologic conditions, which are partly governed by altitude differences among the study sites.

…agricultural production and crops grown in the zone depend on weather conditions and the land settings. Lowland and midland settlers, mainly produce teff, maize, sorghum, and to some extent wheat; whereas highlanders produce barley, wheat and sometimes oats … consumption is largely determined by what is produced.... the exception is that lowlanders produce teff and sell most of it as a cash crop instead of consuming it….

Twenty-eight years, male, agricultural office expert.

Domestic animals, including cows, oxen, sheep, and chicken were owned by most households. A considerable number of households had access to milk and milk products, but have reported that they do not often consume them. The consumption of meat, fish, and poultry, whether pregnant or not, was also rare and was limited to annual religious and new year festivities or special occasions like weddings.

…though livestock rearing is common here, it is rare to eat meat; we do not slaughter animals for household consumption unless the animal is severely ill. On holidays, however, everyone slaughters at least a chicken…

Grandmother, 78 years, claimed to have lived >40 years in the study area.

#### Quantity of food consumed

Although pregnant women are expected to increase the amount of food they consume, they have reported to have reduced their food intake on various occasions. This was mainly ascribed to pregnancy-related discomforts like nausea, vomiting, and morning sickness. Some have also complained of poor appetite and reduced interest for food as a result of gastric irritation and dyspepsia.

… you are asking us to know if we have increased the amount of food we consume per meal during pregnancy,….yet, in many cases, we rather have poor appetite,… we take less food than usual as most of us get sick during pregnancy…

Pregnant woman, 28 years, FGD participant, speaking loud and the remaining participants nodding their heads in favor.

The action taken to cope with the reported gastric irritation was either to avoid specific food groups believed to aggravate their conditions or to decrease/modify their consumption. Among the foods mentioned in this regard are *injera* and hot stews that contain pepper or spice as ingredients.

… we usually eat a less acidic form of injera prepared by reducing the number of days of fermentation (locally known as ‘aflegna’). We also avoid stews containing pepper to avoid gastric irritation …. Some mothers would even skip certain meals to escape the digestive discomforts …

Pregnant women, 31 years, FGD participant.

Such side effects, along with the lack of constant supply of the supplements, could be the reason behind the low compliance and adherence to the IFA supplementation during pregnancy.

…. The nurse at the nearby health center gave me 90 tablets in three rounds, but I couldn’t take more than ten… I suffered from gastric irritation…. I have told the problem to the nurse, but she told me to take it after eating food with ample of water; I didn’t get better…. I thus discontinued taking the pills…. I believe that the tablets would have helped me, but what should I do to the gastric irritation…

Pregnant women, 35 years, diagnosed with anemia.

### Food taboos and perceptions related to weight gain during pregnancy

Divergent opinions were noted regarding the practice of food taboos. Some health workers and key informants believed that food taboos are becoming an old story.

….. Except for few illiterate women that live in remote rural areas, I do not think that many still believe that some foods need to be avoided during pregnancy…

Nurse, female, 34 years, born and working in the study area.

In contrast, a considerable proportion of the elderly and some pregnant women and their husbands still believed that some foods should be avoided during pregnancy.

… pregnant women should be careful and avoid certain foods, particularly towards the last trimester. Our community strongly believes that what a woman eats after her eight months of pregnancy goes directly to the womb to feed the baby. Thus, some foods can hurt the fetus…

Pregnant women, 34 years, FGD participant.

Among foods considered to be taboo are leafy vegetables like cabbage. Some study participants mentioned that in their culture, if a pregnant woman eats leafy vegetables, especially after 8 months of gestation, the leaf passes to the womb and attaches to the baby’s head and form what they called “particles”. These “particles” are considered harmful to the child and are even considered to cause immediate death to the newborn. Similarly, the consumption of dairy products like milk, yogurt, and cheese during pregnancy is considered harmful to the fetus.

….we old people believe that pregnant women should avoid consuming dairy products like yoghurt and cheese, particularly as the gestational age advances. This is because dairy products can pass to the womb and attach to the baby’s head… I have seen this happen with my naked eye: a baby born full of milk products on the head… full of cream and cheesy substances… I have witnessed these babies dying immediately after delivery…

Husband of pregnant women, 74 years, KII respondent.

Similarly, the discussants considered foods like fruits, sugarcane, and some types of vegetables as taboo. The consumption of these foods was also perceived to be associated with having bigger babies, which is believed to lead to a difficult delivery.

… I doubt the effect, but our community strongly believe that if a pregnant woman eats sugar cane, fruits, and some other vegetables, she may have a big baby which endangers her life by making labor difficult…

A focus group discussant, pregnant woman.

Almost all of the pregnant women, their husbands, and the elderly involved in the KIIs and FGDs disfavored weight gain during pregnancy in fear of having big babies that can complicate delivery. They have said that delivering a bigger baby can be life-threatening for both the mother and the newborn.

….I know that no one dislikes to have a big and handsome baby; we in fact feed our babies to make them grow big during early childhood… but in the womb, it is very risky … we thus decrease our food intake to limit the size of the fetus and facilitate its delivery….

Pregnant women, 29 years, FGD participant

## Discussion

The present KIIs and FGDs conducted in four rural districts of Arsi Zone have identified that little change occurs in the diets of the women as they transit from pre-pregnancy to pregnancy. The diets remain predominantly plant based, with little or no consumption of animal-source foods, fruits, and vegetables. The limited production and market availability, the seasonality of production, and the several taboos identified limit the consumption of fruits and vegetables during pregnancy. In addition to the limited dietary diversity, the amount of food consumed are often reduced due to gastrointestinal irritations or intentional food restrictions from fear of a possible obstructed labor associated with the delivery of a bigger baby.

Pregnancy is a critical window of opportunity when the fate of the fetus starts to be decided [16]. Organs are being formed, and the fetus grows at an extremely rapid rate [17]. This all leads to increased nutrient needs that require the pregnant women to increase both the diversity and the amount of foods consumed [8]. Contrarily, pregnant women in the study area reported that their dietary diversity (quality) during pregnancy remained similar or lower than the pre-pregnancy state. This is consistent with an earlier report from a study conducted in West Arsi, Ethiopia [18], and can partly explain the highly prevailing malnutrition observed during pregnancy [19–22]. Such undiversified diets have been associated with adverse pregnancy outcomes including anemia, low-birth weight, and preterm deliveries [23].

Indeed, the inadequate intake of various nutrients, including those of iron, folate, etc. has been closely linked to adverse pregnancy outcomes [24]. Consumption of animal source foods (ASFs), along with fruits and vegetables, can improve the intake of critical micronutrients. However, like in most rural settings in low-income countries, the consumption of ASFs, fruits, and vegetables was rare [25]. The low consumption of fruits and vegetables was partly attributed to the relatively low production and market accessibility of these produces. This is corroborated by the very low (<5 %) national fruits and vegetable production figures [26], which further highlights the need to make the agriculture sector and perhaps the overall food system more nutrition sensitive.

Increasing the production and accessibility of nutrient-dense foods alone would not be enough to improve the nutritional status of the pregnant women in light of the identified food taboos and misconceptions. Most of the identified food taboos, although their reasons are not scientifically supported, seem to have originated from the intention to prevent pregnancy complications. For example, a child may be born with meconium stain as a result of a prolonged and obstructed labor; the associated dark-green color and sometimes grey-whitish particle led to its association with the consumption of vegetables and dairy products during pregnancy, making dairy products and vegetables a taboo. Similar taboos have also been noted in a recent quantitative study conducted in West Arsi [18].

Despite the general consideration that optimal weight gain is indicative of a healthy pregnancy [27], this was disfavored in fear of complicated deliveries. This negative attitude towards weight gain during pregnancy could be related to the higher risk of obstetric complications that may be faced during the delivery of a bigger infant. A risk greatly increased by the short stature and the associated reduced pelvic capacity of the mothers [28]. However, such inadequate food intake compounded with the very low compliance to the routine IFA supplementation may lead to intrauterine growth restriction and low-birth weight [29].

In line with earlier findings from studies conducted in Ethiopia [18] and elsewhere [11–13], food taboos were found to be dominant among remote rural residents with little access to nutrition and health services, older, and uneducated women. In such circumstances, cultural believes, whether right or wrong, tend to shape behaviors. However, this finding also shades hope that such misconceptions and taboos may gradually be circumvented with increased access to education and health services.

Several limitations need to be considered when interpreting our findings. First, although the KIIs and FGDs were carefully conducted, we do not know to what extent women over-reported or underreported positive or negative behaviors. While the villages were randomly selected, they may not represent the 22 districts found in Arsi Zone. Besides, the sampling of key informants and focus group discussant was purposive. Although food taboos and food restrictions were identified as a problem in this community, their practice may depend on the age of study participants, their educational status, location of residence, etc. Thus, the findings should not be generalized to the whole study population and beyond, but should be taken as an indication that such practices are still present among some of the studied subjects.

## Conclusion

The present study has shown that the dietary quality of the pregnant women remains as low as, or sometimes lower than the pre-pregnancy state, partly because of the limited access/availability of ASFs, fruits, and vegetables. This, along with the prevailing food taboos and misperception towards weight-gain during pregnancy could lead to adverse pregnancy outcomes. Interventions that diversify agricultural production to make fruits and vegetables more accessible, along with educational interventions emphasizing on the importance of adequate food intake during pregnancy, both in terms of quantity and quality, are needed. Such interventions may benefit from correcting the prevailing misconceptions, improving the health infrastructure in rural settings, and encouraging pregnant women to deliver their babies in a health facility, where help can be available in case of pregnancy complications.

## Abbreviations

APHRC, African Population and Health Research Center; ASF, animal source foods; FGD, focus group discussions; IDRC, International Development Research Center; IFA, iron folic acid; KII, key informant in-depth interviews; LMIC, low- and middle-income countries; NGO, non-government organization
